# Electronic application for rabies management improves surveillance, data quality, and investigator experience in Haiti

**DOI:** 10.3389/fvets.2023.1052349

**Published:** 2023-03-31

**Authors:** Caroline A. Schrodt, Pierre Dilius, Andrew D. Gibson, Kelly Crowdis, Natael Fénelon, Yasmeen Ross, Sarah Bonaparte, Luke Gamble, Frederic Lohr, Haïm C. Joseph, Ryan M. Wallace

**Affiliations:** ^1^National Center for Emerging and Zoonotic Infectious Diseases, Centers for Disease Control and Prevention, Atlanta, GA, United States; ^2^Epidemic Intelligence Service, CDC, Atlanta, GA, United States; ^3^Haiti Ministry of Agriculture, Rural Development and Natural Resources, Port au Prince, Haiti; ^4^Mission Rabies, Cranborne, Dorset, United Kingdom; ^5^The Royal Dick School of Veterinary Studies and The Roslin Institute, University of Edinburgh, Edinburgh, United Kingdom; ^6^Christian Veterinary Mission, Port au Prince, Haiti; ^7^Pan American Health Organization, Port au Prince, Haiti

**Keywords:** rabies, Haiti, integrated bite case management, one health, surveillance, electronic application, tablet, smartphone

## Abstract

**Background:**

Integrated bite case management (IBCM) is a multi-sectoral response to animal-bites which reduces human and canine rabies mortality through animal quarantine, bite-victim counseling, and vaccination tracking. Haiti's national rabies surveillance program was established in 2013 using paper-based IBCM (pIBCM) with adoption of an electronic smartphone application (eIBCM) in 2018.

**Methods:**

We evaluated the feasibility of implementing the electronic app in Haiti and compared pIBCM and eIBCM data quality collected January 2013–August 2019. Deaths prevented, cost-per-death averted, and cost-per-investigation during use of pIBCM and eIBCM were estimated using a previously validated rabies cost-effectiveness tool that accounted for bite-victim demographics; probability of acquiring rabies; post-exposure prophylaxis; and costs including training, supplies, and salaries. We compared pIBCM and eIBCM based on data comprehensiveness, completeness, and reporting efficiency. Surveys were administered to IBCM staff to evaluate the usefulness, simplicity, flexibility, and acceptability of eIBCM.

**Results:**

Of 15,526 investigations, 79% were paper-based and 21% electronic. IBCM prevented 241 (estimated) human rabies deaths. Using pIBCM, cost-per-death averted was $2,692 and the cost-per-investigation was $21.02; up to 55 data variables were collected per investigation; data transmission took 26 days to reach national staff, and 180 days until analysis. Using eIBCM, the cost-per-death averted was $1,247 and the cost-per-investigation was $22.70; up to 174 data variables were collected per investigation; data transmission took 3 days to reach national staff, and 30 days until analysis. Among 12,194 pIBCM investigations, 55% were mappable by commune, compared to 100% of eIBCM investigations mappable by GPS. Animal case definitions were incorrectly ascribed by investigators in 5.5% of pIBCM investigations and zero for eIBCM; typically, errors were in determining probable vs. suspect case assignments. Overall, eIBCM was well-accepted by staff, who reported the app is easy-to-use, facilitates investigations, and compared to pIBCM hastens data reporting.

**Discussion:**

In Haiti, eIBCM showed improved data completeness, data quality, and shorter notification times with minimal increase in operational cost. The electronic app is simple-to-use and facilitates IBCM investigations. Rabies endemic countries could refer to eIBCM in Haiti as a cost-effective means to reduce human rabies mortality and improve surveillance capacity.

## Introduction

Rabies is a highly lethal virus that is considered universally fatal among those who develop clinical signs and symptoms (1). Despite highly effective vaccines, 59,000 annual human deaths from rabies are estimated to occur world-wide, with 99% of deaths attributed to exposures from dog bites (2, 3). In countries with effective canine rabies vaccination and surveillance programs, coupled with ample availability of post-exposure prophylaxis (PEP), human rabies case fatality rates are drastically reduced (4). In the Western Hemisphere, Haiti is one of several countries that have not yet achieved effective canine rabies control and continue to report human deaths due to rabies (5–7). Whilst the true incidence of human rabies in Haiti remains unknown, a 2015 global rabies burden study estimated 130 human rabies deaths occur annually; an estimate which has likely been reduced by 50–65% since the implementation of a national Integrated Bite Case Management program (8–11).

Integrated Bite Case Management (IBCM) is recommended by the World Health Organization (WHO) for passive rabies surveillance (12). Typical IBCM programs rely on routine communication between healthcare providers who treat bite victims, and veterinary professionals who investigate animals suspected to have rabies. Under an ideal IBCM program, bites are immediately reported to veterinary professionals who initiate field investigations. Several publications have shown that the combined actions of risk assessments, patient counseling, dog quarantine, and sample collection and testing can greatly reduce the risk of human rabies deaths and can be implemented in manners that are highly cost-effective (8).

In 2011, the Haiti Animal Rabies Surveillance Program (HARSP) was created to improve rabies diagnostic laboratory testing capacity, train animal surveillance officers, and improve routine animal surveillance (13). In 2013, IBCM investigations began under HARSP, further building the framework for rabies management in Haiti through community animal bite and rabies investigations. Data during an IBCM investigation is collected by the investigators, reported to the treating healthcare provider and national animal health officials, and analyzed by the national program. In addition to collecting data for surveillance purposes, the bite case investigations identify sick animals and bite victims (human and animal) and facilitate testing or treatment as appropriate. In 2018, after realizing that paper-based surveillance forms were suffering from high data entry error rates and lacking variables necessary for programmatic monitoring and evaluation, the IBCM investigations converted from paper-based forms to a cell-phone or tablet-based application (“app”) to facilitate case investigations and collect data simultaneously.

The REACT app is now used in eight countries, is available in five languages and has recorded over 40,000 notifications of suspect rabid animals; highlighting both the need for improved rabies surveillance capacity globally and the versatility of electronic tools to be adapted for use and implemented in a variety of low-resource rabies endemic settings (14). IBCM continues to provide a framework for bite case investigations and surveillance in Haiti, which is crucial for a One Health approach in combatting dog-mediated rabies.

Here, we describe the implementation of a national electronic IBCM (eIBCM) program in Haiti using a Rabies Exposure Assessment and Contract Tracing (REACT) app. We evaluated paper-based IBCM (pIBCM) and eIBCM to estimate the number of human deaths averted, costs, quality of data outputs, and user acceptability to determine the feasibility of introducing the electronic REACT app in low-resource settings.

## Methods

The REACT app is developed and supported by the Worldwide Veterinary Service and is available on both Android and iOS operating systems (14). Investigators surveyed during this study used Android REACT versions 1.0–1.3 on handheld Samsung Galaxy Tab A T285 tablets. The REACT app interfaces with a secure cloud-based server and backend system which is accessed via password-protected logins by project managers. The REACT app is organized into five sections: (1) Event Notification, (2) Animal Health Investigation, (3) Rabies Exposure Investigation, (4) Animal Quarantine, and (5) Test Results. Each section has standardized data collection forms with limited open-text fields. REACT provides in-app guidance to investigators, such as the rabies risk status or case assignment of the animal, recommended quarantine schedules, and prompts to complete critical data fields. REACT is currently available in English, French, Creole, Spanish, and Vietnamese.

To evaluate the performance of IBCM in Haiti, we applied the U.S. Centers for Disease Control (CDC) Morbidity and Mortality Weekly Report (MMWR) Updated Guidelines for Evaluating Public Health Surveillance Systems (15). Quantitative and qualitative attributes (cost-effectiveness, timeliness, data quality, usefulness, simplicity, flexibility, acceptability, and stability) were evaluated by reviewing current methods/protocols as well as results derived from a survey administered to all IBCM investigators.

**Figure 1:**
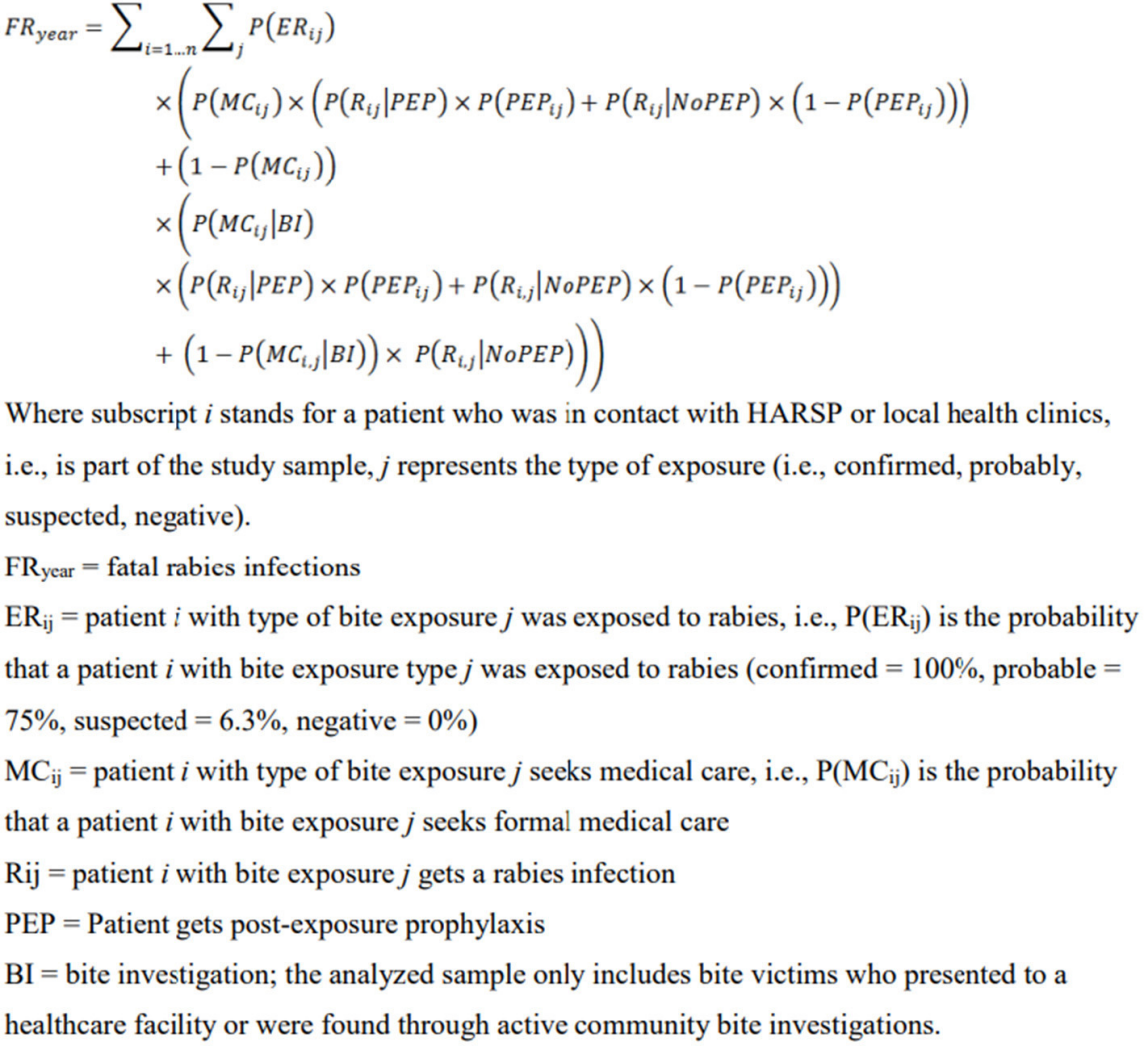
Fatal rabies infections model excerpt from Undurraga et al., “Cost-Effectiveness Evaluation of a Novel Integrated Bite Case Management Program for the Control of Human Rabies, Haiti 2014–2015” Supplementary material.

Data were evaluated from rabies IBCM data collected by the Haiti Ministry of Agriculture and Rural Development from January 2013–August 2019. We assessed the feasibility of HARSP to determine if the program is locally viable and pragmatic by estimating key economic indicators for program operation. The number of human deaths prevented, cost per death averted, and cost per investigation were calculated separately for pIBCM and eIBCM and were estimated using a validated, evidence-based Rabies Cost-Effectiveness Tool developed by Undurraga et al. (11) in Microsoft Excel. Deaths averted were calculated compared to a non-IBCM (NBCM) rabies management program (Supplementary material; pIBCM & eIBCM Ecoomic Analyses). This tool applies a probabilistic model (Figure 1) to estimate deaths prevented utilizing IBCM-collected data on number of bite-victims, probability of acquiring rabies stratified by the case outcome of the animal, probability of initiating post-exposure prophylaxis, and the probability of dying from rabies in the absence of PEP. Cost factors included training, supplies, staff, and salaries (Supplementary material; pIBCM & eIBCM Economic Analyses). Differences among input parameters are highlighted in Table 1. We compared three aspects of surveillance data quality captured through pIBCM and eIBCM that correspond to the following MMWR evaluation categories: data comprehensiveness (total number of data fields available), data completeness (automatic variable, location, and animal case definition assignments), and reporting efficiency (time from data entry to reporting and data analysis ascertained from programmatic timelines).

**Table 1:** Differences among input parameters for pIBCM and eIBCM.

**Microsoft excel tab**	**Variable**	**Units**	**pIBCM**	**eIBCM**
HARSP_data	Study population	N	12,194	3,332
	Time frame for analysis	Years	5.6	1.1
	Share of PEP treatment paid for by the government	%	50%	50%
	Human exposures to rabies			
	Confirmed	N	257	23
	Probable	N	892	794
	Suspected	N	2018	1099
	Negative	N	9362	1317
HARSP$Surv^*^	Tablet + Sim card	$/worker	0	145
HARSP$Train	Classroom days	N	5	3
	Field days	N	5	3
	Form training	N	1	2
	Number of participants	N	20	10
	Days in training destination	N	12	8
	Salary/wage	$/day	12	8
	Travel expenses (per diem, hotel)	$/day	12	8
Dog_invest	Dog-investigations	N	12,194	3,332
	Confirmed rabid	N	170	30
	Probable rabid	N	630	488
	Active surveillance	N	34	0
	Diagnosed	N	27	0
	Confirmed	N	3	0
	Passive surveillance (located & non-located)	N	12,160	3,332
	Non-located	N	2,100	1,153
	Probable	N	355	220
	Located	N	10,060	2,179
	Dogs investigated and found dead	N	517	110
	Confirmed rabid	N	95	27
	Probable rabid	N	129	61
	Dogs investigated and found alive	N	9,543	2,069
	Dogs immediately euthanized	N	121	3
	Confirmed rabid	N	35	2
	Probable rabid	N	5	0
	Dogs under observation	N	9,209	1,853
	Confirmed rabid	N	36	1
	Probable rabid	N	115	9
	Dogs quarantined	N	6	0
	Confirmed rabid	N	1	0
	Probable rabid	N	0	0
	Evaded capture	N	207	213
	Probable rabid	N	22	198

^*^Cost per vehicle for both pIBCM and eIBCM was $1200 which represents a $200 increase from the original model and reflects the increase in vehicle cost over time.

Two survey versions were used, the first for staff who used both paper investigation forms and the REACT app (Survey 1.0), and the second for staff who only used the eIBCM app (Survey 2.0) (Supplementary material). Surveys were written in English and provided to Ministry of Agriculture, Natural Resources, and Rural Development (MARNDR) officials in Haiti, where they were adapted for local use by translating the surveys into local languages and reviewing them for comprehensibility. Each survey gathered data from the staff including demographics, years of experience working with HARSP, and perceptions regarding use of the REACT app and paper investigation forms for those who were employed by MARNDR from 2013 to 2019. The national program manager administered the survey in French or Haitian Creole during phone interviews with HARSP staff from April 6 to April 29, 2020. Survey 1.0 had 35 questions and Survey 2.0 had 34 questions. To evaluate qualitative attributes from the CDC MMWR Updated Guidelines for Evaluating Public Health Surveillance Systems, interviewees were read aloud a statement and asked to indicate the degree of agreement or disagreement using a typical five-point Likert scale (15) (Supplementary material). For the analysis, the answer “Strongly agree” received 5 points, “Agree” received 4 points, “Neither agree nor disagree” received 3 points, “Disagree” received 2 points, and “Strongly disagree” received 1 point. Average response values were calculated for the two groups and compared using a two-tailed independent *t*-test.

## Results

**Figure 2:**
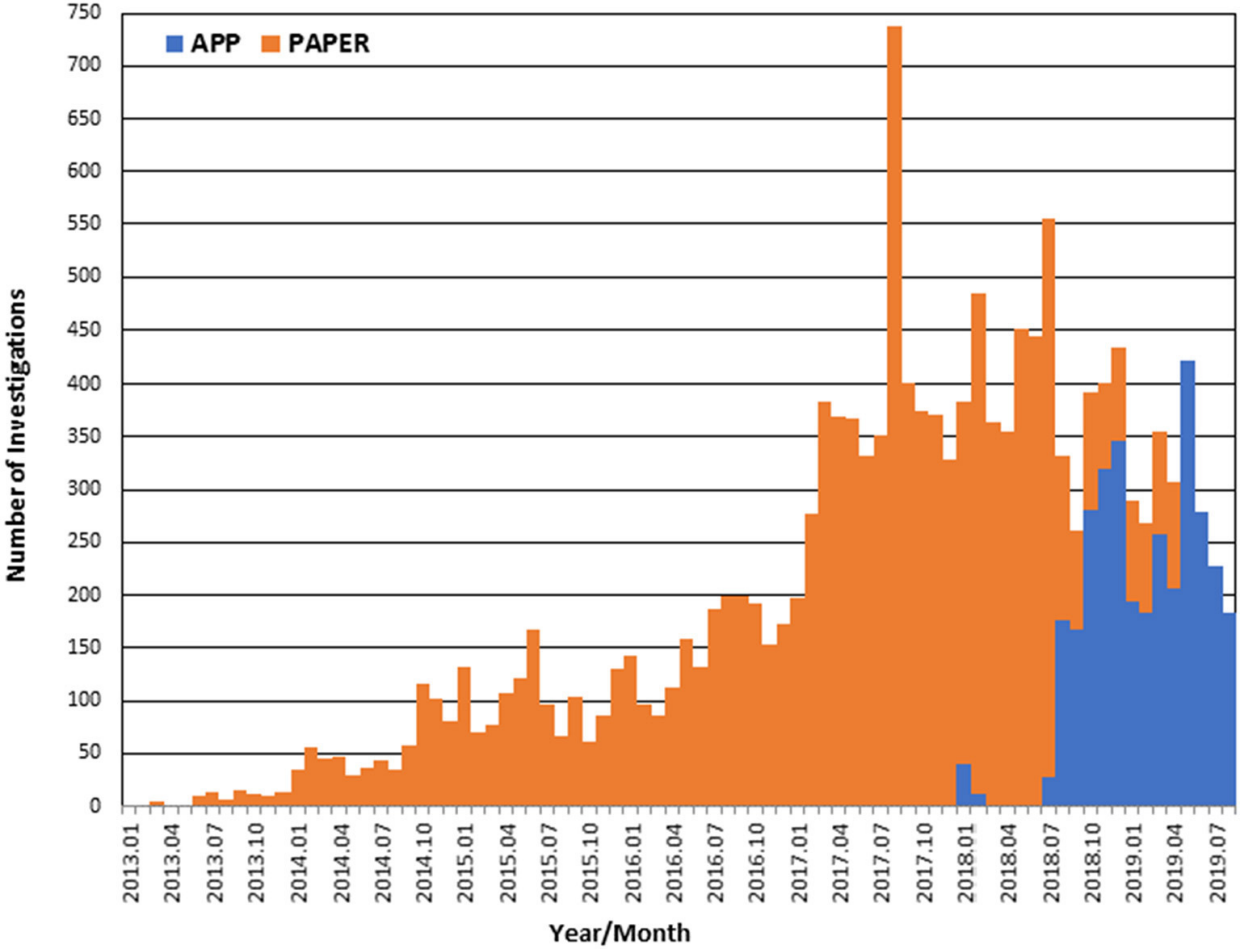
Transition from paper to app-based IBCM, 2013–2019.

**Figure 3:**
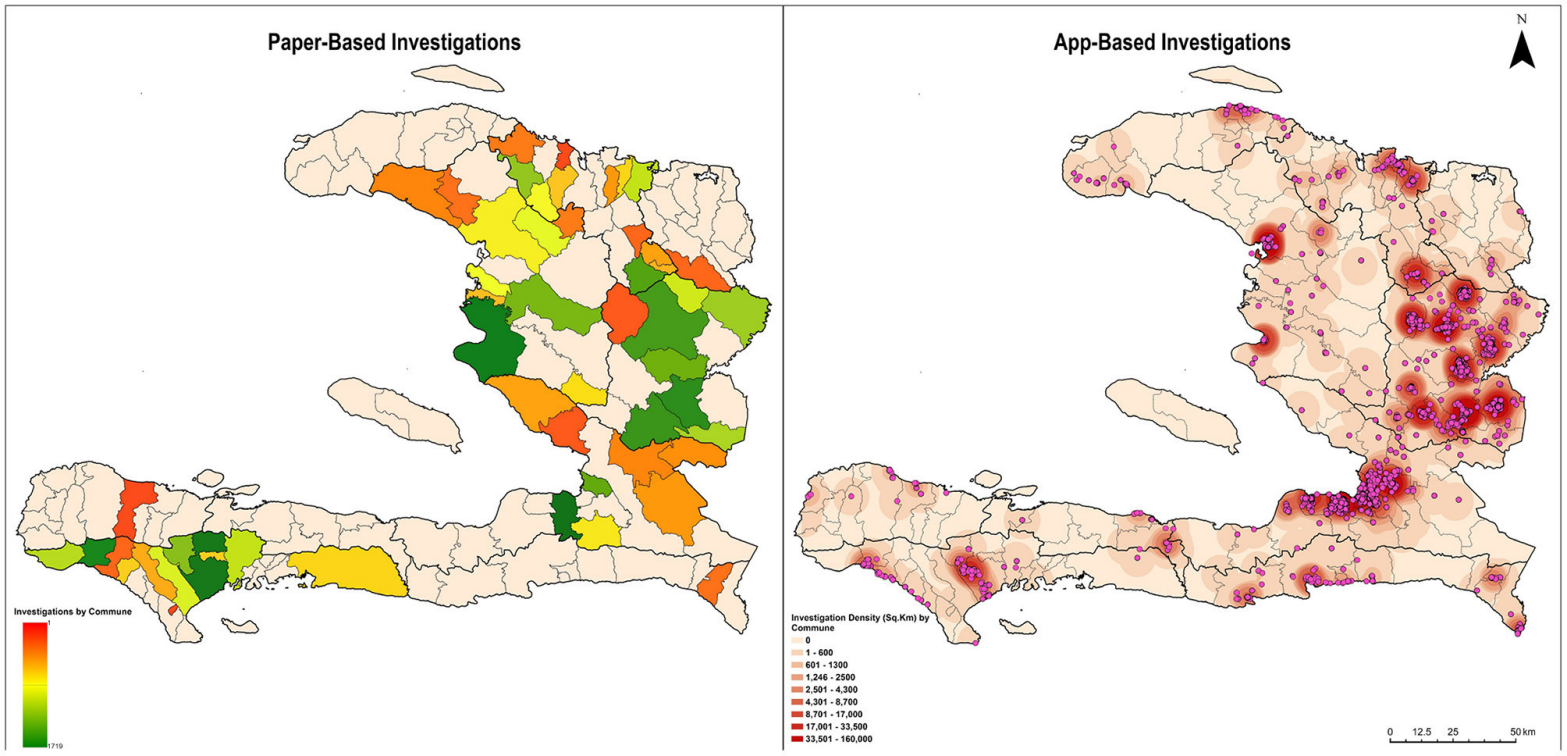
Location and Density of Rabies Case Investigations, Haiti, 2013–2019. Paper-Based Investigations: Total investigations: 12,194; Total mappable investigations: 6,695 (55%) Bite cases are mapped at the level of communes. *Spelling errors in handwritten paper-based investigations did not allow mapping of all investigations. App-Based Investigations: Total investigations, all mappable: 3,332 (100%). Bite cases are mapped using GPS coordinates.

From January 2013 to August 2019, there were 15,526 bite case investigations conducted in Haiti, of which 79% (*n* = 12,194) were paper-based and 21% (*n* = 3,332) were electronic (Figure 2). The REACT eIBCM app was introduced in January 2018 but was not fully recommended to be used by all staff until August 2018. From January to August 2018, an average of 12 eIBCM cases were recorded monthly. After August 2018, an average of 250 eIBCM cases were recorded monthly. From August 2018 until the end of the evaluation period (13 months), most cases were investigated by the eIBCM method (79%) compared to the pIBCM method (21%). The Rabies Cost Effectiveness Tool outputs estimate that, compared to a NBCM rabies management program, 170 human rabies deaths were prevented from January 2013 to August 2019 as a result of HARSP, or that one human life was saved for every 91 investigations. Investigations were performed in 100% (10/10) of departments, and 53% (76/144) of communes throughout the country (Figure 3). All communes are not represented because bite cases were not reported from all locations.

### Evaluation of cost-effectiveness, timeliness, and data quality

During the 67 months in which pIBCM was the primary investigation method, an annual average of 30 cases were laboratory confirmed, 113 were clinically confirmed (probable), the cost per death averted was $2,692, and the cost per investigation was $21.02 (Supplementary material; pIBCM Economic Analysis). During the 13 months in which eIBCM was the primary investigation method, an annual average of 27 cases were laboratory confirmed, 444 were clinically confirmed (probable), the cost per death averted was $1,247, and the cost per investigation was $22.70 (Supplementary material; eIBCM Economic Analysis).

**Figure 4:**
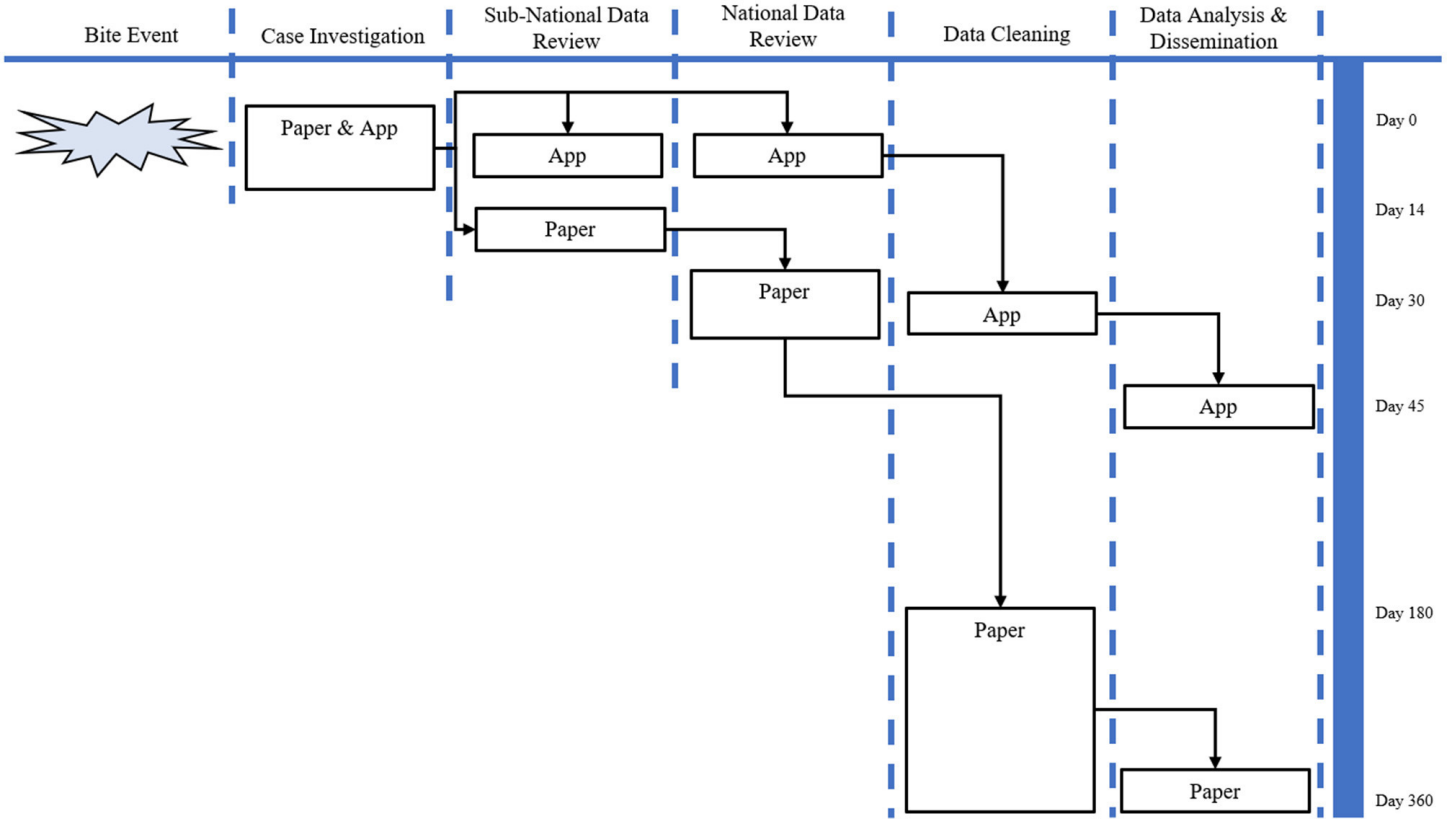
Programmatic flow chart of pIBCM vs. eIBCM.

The number of days from investigation onset to notifying national animal health officials was up to 26 days when using pIBCM compared to only 3 days when using eIBCM *via* the REACT app. The number of days from investigation to analysis was up to 360 when using pIBCM and up to 45 days when using the REACT app (Figure 4). The pIBCM form had 55 data variables, whereas the REACT app had 174 data variables. Unlike pIBCM, eIBCM automatically assigns the user's name and animal ID, collects GPS coordinates, date of investigation, and assigns an animal case status, reducing entry errors and data cleaning requirements (Table 2). Among pIBCM case investigations, 55% (6,695) were mappable at the commune level without requiring extensive data cleaning of hand-written locality information, whereas 100% of eIBCM investigations collected GPS coordinates and were readily mappable (Figure 3). Among pIBCM case investigations, 94.5% (11,526) were determined to have correct animal case definition assignments (Table 3). Of the 5.5% (668) incorrectly assigned, the risk was under-stated for 29.2% (195) case investigations and over-stated for 70.8% (473) case investigations (Table A, Supplementary material).

**Table 2:** Surveillance system attributes.

**Evaluation factor**	Definition^*^	**Assessment Criteria**	**pIBCM**	**eIBCM**
Feasibility	*Feasibility standards ensure that the evaluation is viable and pragmatic. Differing political interests of those involved should be anticipated and acknowledged. The use of resources in conducting the program should be prudent and produce valuable findings*.	Human Deaths Prevented (Annual)^**^	20	55
		Program Operational Cost (Annual)	$45,772	$68,745
		Cost per Death/Disability-Adjusted Life Year Averted	$2,692	$1,247
		Cost per Investigation	$21.02	$22.70
Usefulness	*The program contributes to the prevention and control of adverse health-related events, including an improved understanding of the public health implications of such events. Surveillance data should be useful in contributing to performance measures, including health indicators*.	Frequency of Data Publications	1.4 per year	2.7 per year
		Frequency of Summary Reports Submitted to Relevant Stakeholders	Annually (1 per year), manually produced	Monthly (12 per year), automated in R
Timeliness	*The speed between steps in a public health surveillance system*.	Number of Programmatic Steps from Data Collection until Analysis	7	4
		Time to Complete Data Collection (per data field)	10 s	6 s
		Time from Collection Until Data Review	26 days	3 days
Data Quality	*The completeness and validity of the data recorded in the public health surveillance system*	Key Variables- User, Animal ID, GPS coordinates, Date of Investigation, Animal Case Status	Requires manual entry or retrospective cleaning	Automatically collects
		Investigations mappable without cleaning	55%	100%
		Case Outcomes Correctly Classified (%)	94.5%	100%
Flexibility	*Surveillance system can adapt to changing information needs with little additional time, personnel, or allocated funds. Flexible systems can accommodate new health-related events, changes in case definitions or technology, and variations in funding or reporting sources. In addition, systems that use standard data formats (e.g., in electronic data interchange) can be easily integrated with other systems*.	Number of Data Variables Collected	Up to 55	Up to 174
		Additional Cost per Investigation for eIBCM	Reference	+$1.68
		Data Format	Paper—manual entry required for integration	.csv file format merges with all major software
Acceptability	*The willingness of persons and organizations to participate in the surveillance system*	Average User Rating	Not assessed	97%
		User Preference	0%	100%
Stability	*The reliability (i.e., the ability to collect, manage, and provide data properly without failure) and availability (the ability to be operational when it is needed) of the public health surveillance system*.	Frequency of Lost Data Records	Unable to assess	8% of users reported losing app data at least one time
		Frequency of Inoperable Data Collection Tools	0%	26% of users reported a tablet malfunction at least one time
		Average Delay in Availability of Data to the National Animal Health System	26 days	3 days

^*^Adapted from: U.S. Centers for Disease Control (CDC) Morbidity and Mortality Weekly Report (MMWR) Updated Guidelines for Evaluating Public Health Surveillance Systems.

^**^Deaths prevented should not be directly compared, as this is often a function of changing epidemiology, rabies risks, and fluctuations in staff employed by the program. These numbers represent deaths prevented as compared to a non-IBCM (NBCM) rabies management program.

**Table 3:** Comparison of interim and final animal case status assignments, pIBCM^§^.

**Investigator interim animal case assignment**		**Actual animal case assignment**			
		**Confirmed**	**Probable**	**Suspect**	**Non-case**
Confirmed	185	**178**	3^*^	4^*^	0
Probable	629	0	**179**	442^*^	8^*^
Suspect	1.943	0	61^†^	**1,868**	14*
Non-case	9.362	0	14^†^	47^†^	**9,301**
Unassigned	75	0	22^†^	51^†^	2^*^
TOTALS	12.194	178	279	2,413	9,325
% Concordance		100%	64.2%	77.4%	99.7%

^*^Over-stated interim risk.

^†^Understated interim risk.

^§^There are no investigator-assigned animal case assignments when using eIBCM because the app automatically assigns the animal case status.

Bold text denotes concurrence between the interim animal case assignment by the investigator and the actual animal case assignment as determined by final data review.

### IBCM investigator assessment

Thirty-three past and current IBCM investigators and program managers completed the eIBCM satisfaction survey. Of these, 19 conducted pIBCM before transitioning to eIBCM and were administered Survey 1.0; the remaining 14 had only ever performed eIBCM and were administered Survey 2.0 (Supplementary material). Respondents consisted of 23 IBCM investigators, nine departmental managers, and the national manager. The mean number of years interviewees worked with HARSP was 2.2 years (26.7 months), ranging from 0.1 to 7.2 years (1 to 86 months).

Investigators self-reported that they were proficient with the REACT app after an average of 5 investigations (95% CI 4.2–5.7 investigations) (range 2–10 investigations). On average, users reported that it took 17 min (95% CI 14–21 min) to complete a case investigation report in the REACT app, or 5.9 s per data field. Investigators who used paper investigation forms reported that it took an average of 5 min (95% CI 4–6 min) to complete the paper case investigation report, or 10 s per data field. Among 32 of the interviewees, 22 reported taking paper notes during the investigation and entering data into the REACT app at a later time, whereas the remaining 10 reported entering data directly into the REACT app at the time of the investigation.

**Figure 5:**
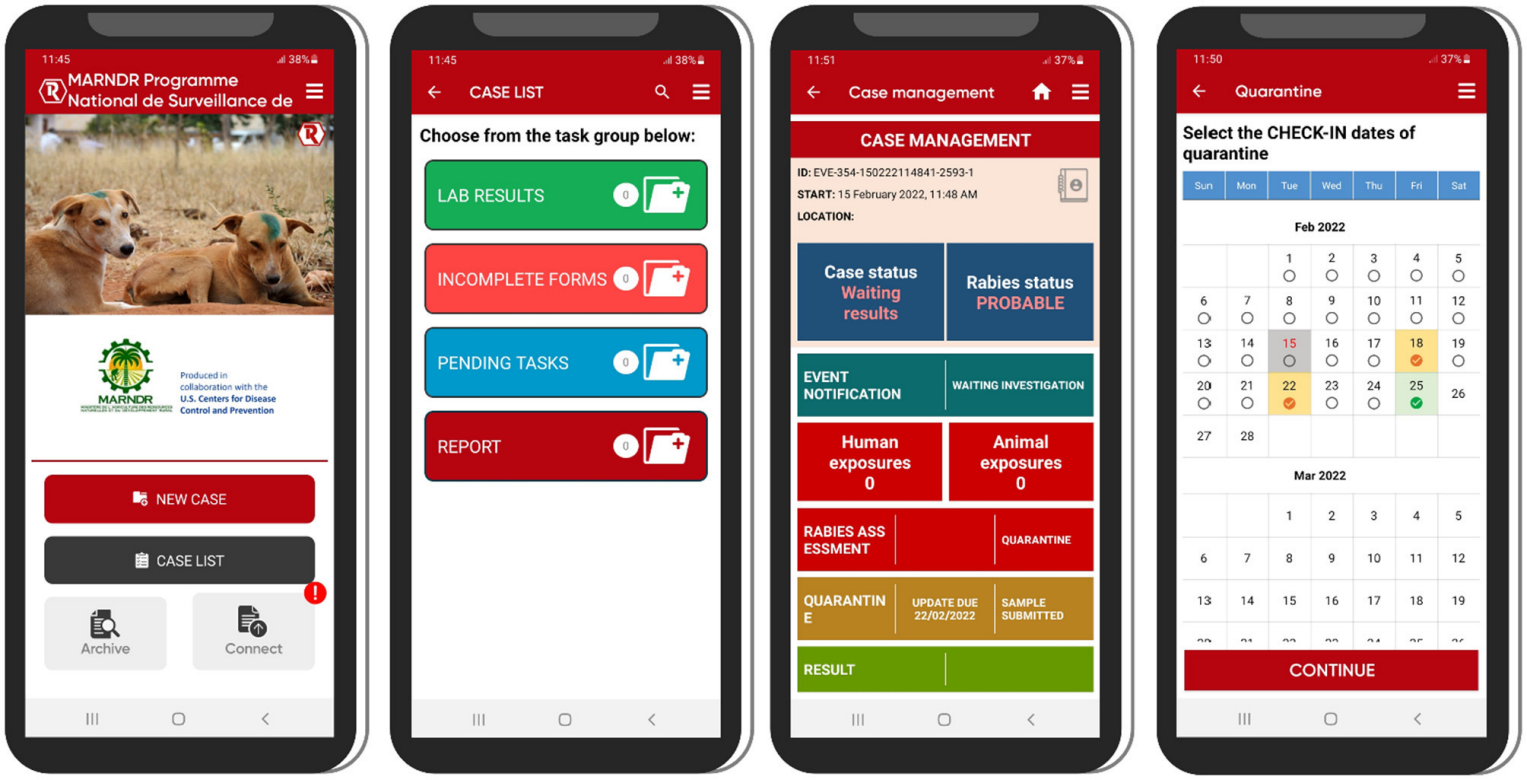
Examples of the user interface for the REACT app on Android devices. Left to right: Home screen with navigation buttons; Case list screen for navigating to pending cases; Case management screen for management of a specific case; Quarantine scheduling screen for scheduling follow-up actions during an animal's quarantine period.

Every user reported encountering at least one problem while using the REACT app, although this occurred rarely for the majority of users (61%) (Table 4). Issues encountered while using the REACT app were primarily due to infrastructural limitations including no internet access (11% “never,” 67% “rare,” 19% “sometimes,” 4% “often”), tablet battery died during data entry (38% “never,” 52% “rare,” 5% “sometimes,” 5% “often”), and no access to electricity to charge the tablet (95% “never,” 5% “often”). App-related issues were also reported, including users indicating that the app was too difficult to use (95% “never,” 5% “rare”) and that the app malfunctioned during data entry (63% “never,” 30% “rare,” 4% “sometimes,” 4% “often”). Survey respondents also expressed frustration due to incomplete translation from English to Creole for certain app modules (e.g., the Home Screen) (Figure 5).

**Table 4:** Frequency of problems experienced while using REACT smartphone application for investigation of suspected human rabies exposures, Haiti, 2020.

**eIBCM Complications**, ***n*** = **33**				
Infrastructure-related	Never (%)	Rare (%)	Sometimes (%)	Often (%)
No internet	11	67	19	4
No electricity to charge	95	0	0	5
Battery died during data entry	38	52	5	5
Tablet is too difficult to use	100	0	0	0
Tablet is lost or stolen	100	0	0	0
Tablet is broken	61	29	11	0
App-related				
App is too difficult to use	95	5	0	0
App malfunction	63	30	4	4
Any problem	0	61	25	14

On average, interviewees agreed or strongly agreed with statements assessing the REACT app in terms of ease of use [4.9 for Paper and App (P-A) Investigators; 4.7 for App-only (A) Investigators; *p* < 0.01], timeliness of report submission (4.9 P-A; 4.7 A), investigation thoroughness (4.8 P-A; 4.9 A), rabies risk assessment (4.8 P-A; 4.3 A; *p* < 0.01), case determination (4.8 P-A; 4.6 A), quarantine period determination (4.9 P-A, 5.0 A), communication with bite victims (4.9 P-A; 4.8 A), and timeliness of data analysis (4.7 P-A; 4.2 A; *p* < 0.01) (Table 5). All the interviewees agreed that the REACT app should be the primary method of data collection under the HARSP.

**Table 5:** Comparison of investigator assessment of the REACT smartphone application for investigation of suspected human rabies exposures, Haiti, 2020.

**Assessment criteria**	**P-A investigators^a^**	**A investigators^b^**	***P*-value^c^**
Easy to submit reports	4.9 (4.8–5.0)	4.7 (4.4 – 4.9)	< 0.01
Fast to submit reports	4.9 (4.7–5.0)	4.7 (4.4–4.9)	0.11
Facilitates thorough investigations	4.8 (4.7–5.0)	4.9 (4.7–5.0)	0.75
Helps assess rabies risk	4.8 (4.6–5.0)	4.3 (4.1–4.6)	< 0.01
Helps assess case determination	4.8 (4.6–5.0)	4.6 (4.3–4.9)	0.32
Helps determine quarantine periods	4.9 (4.7–5.0)	5.0 (5.0–5.0)	0.15
Helps communication to bite victims	4.9 (4.8–5.0)	4.8 (4.5–5.0)	0.36
Facilitates timely data analysis	4.7 (4.5–4.9)	4.2 (3.9–4.5)	< 0.01
Total score (Maximum 40 points)	38.8 (37.9–39.6)	37.1 (35.9–38.4)	0.04

^a^Investigators who investigated rabid animals using the paper form, before transitioning to REACT app, *n* = 18.

^b^Investigators who have only ever used REACT app, *n* = 15.

^c^two-tailed *P*-value based on two-sample independent *t*-test.

## Discussion

Rabies control in Haiti has been challenged by earthquakes, hurricanes, the COVID-19 pandemic, and political disruptions, yet the system has remained operational (16, 17). Neglected diseases are often ignored because of poor data quality that results in limited visibility to the true burden of disease (18). REACT offers a way to both increase case detection and improve data dissemination, offering a potential means of overcoming these systemic barriers in the control of neglected diseases. Compared to pIBCM, eIBCM is also cost-effective, has improved data quality, and facilitates more rapid data analysis and dissemination. As the REACT app has been used by all HARSP investigators with positive feedback, eIBCM in Haiti complies with the surveillance evaluation criteria as simple, flexible, and acceptable.

The increased cost per investigation associated with the REACT app is nominal compared to the paper-based system, and is clearly outweighed by the unique benefits offered by the app. The only costs required for eIBCM-specific investigations were for tablets, data, and training, which amounted to < $300 per person-year. The cost per death-averted in both pIBCM and eIBCM were less than reported previously in Haiti by Underraga et al. in 2014 and 2015, who reported estimated ranges of $2,891–$4,735 and $3,534–$7,171 (11), and nearly three times lower than the cost effectiveness threshold set by the WHO (19). This difference can be attributed to changes in programmatic operations and a changing epidemiologic landscape; cost per death averted is heavily influenced by the proportion of high-risk cases that are investigated, which can change through both natural cycles as well as interventions (e.g., vaccination programs).

The differences noted in this evaluation between pIBCM and eIBCM cost per death averted likely reflect normal temporal variation in rabies risks and fluctuation in the number of investigators employed by HARSP. The economic model is sensitive to the proportion of investigation outcomes resulting in confirmed and probable rabid animals, which can change over time due to natural and surveillance operational factors. At the time of eIBCM implementation, HARSP underwent budget cuts resulting in the loss of half of investigators. A drop in operational costs resulted, as well as a noticeable decline in case investigations, as seen in Figure 2. Furthermore, the proportion of high-risk investigations increased from 9% during pIBCM to 24% during eIBCM, reflecting a combination of reduced staffing and an increase in rabies transmission across Haiti. These factors are difficult to control in low-and-middle income countries and highlight the difficulty of utilizing cost-effectiveness measures to compare programs operated in different time periods. Regardless of the operational and epidemiological changes over the 6 years of this program, pIBCM and eIBCM programs were both highly cost-effective per standards established by WHO.

In Haiti, eIBCM showed improved data completeness, data quality, and a shorter notification time compared to pIBCM. Improved data quality and shorter time to analysis allows program managers to identify trends and react more quickly to urgent events. Investigations using pIBCM are only readily mappable at the level of the commune, introducing bias in surveillance data. The automatic collection of GPS coordinates with eIBCM helps investigators accurately evaluate the program, monitor geographic trends, and focus control measures. Electronic IBCM expedites the availability of field rabies surveillance data to health officials and raises real-time awareness of outbreaks. For example, from July to December, 2018, a rabies outbreak was detected in a Dominican Republic city which borders Haiti. No data was available in the Haitian border-city to determine if the outbreak had spilled into Haiti. In January 2019, a bi-national dog vaccination program was conducted and the REACT app was deployed in this Haitian city to monitor rabies exposures (20). By June 2019, 26 rabies investigations were conducted and no dogs had signs consistent with rabies, affirming that the mass vaccination campaign had effectively halted rabies transmission in these two cities (20). Since July 2021, REACT has implemented monthly rabies reports to improve early outbreak detection, detailing location of cases, investigator activity, and laboratory results (Supplementary material). Timely reporting of surveillance data enables program managers to make informed public health decisions and allocate resources based on the changing epidemiology of the disease, allowing for better management of field staff and improving stakeholder engagement.

Following a bite event or reports of suspected rabid animals, timely IBCM investigations can result in a myriad of benefits (8, 13, 21). People exposed are identified more quickly and directed to appropriate medical care, resulting in improved patient outcomes (8). Rapid identification and removal of suspected animals prevents additional bite exposures to people or animals and interrupts the enzootic transmission cycle in dogs (13, 21). However, these benefits are dependent on a well-trained workforce that understands the risk assessment process, PEP recommendations, and quarantine guidance. Our evaluation found that risk assessment determinations from field investigators were prone to some degree of error, resulting in several hundred bite victims receiving incorrect risk counseling. Over-stating the risk in the biting animal can result in unnecessary PEP that can lead to unnecessary medical costs and can diminish oft-limited human vaccines. Conversely, understating the risk could lead to reduced compliance with the PEP regimen and put human lives at risk. The REACT app automatically applies the WHO case status definition for each animal under investigation (confirmed, probable, suspect) and assigns the appropriate quarantine recommendation based on data inputs. The automated algorithms prevent user misassignment of animals, ensuring the appropriate human rabies post-exposure prophylaxis recommendations are communicated. Automated case classification also improves timeliness of data analysis, as these case-by-case determinations do not need to be validated manually as was necessary under pIBCM. The ability of eIBCM to automatically interpret rabies case classifications, while incredibly important from an operational viewpoint, was also greatly appreciated by the investigators.

Mobile electronics are increasingly used for medical and public health purposes and, as of 2020, 93% of the world's population has access to mobile broadband networks (22, 23). However, few smartphone/tablet apps have been used in the surveillance, management, and prevention of rabies. Previous electronic apps used in Tanzania and Haiti, including a component of the app described in this paper, have been used for counting and geographic tracking during dog vaccination campaigns (24, 25). Additional data platforms and apps have been used in Sri Lanka, Tanzania, and Pakistan to monitor human rabies cases, notify and track persons receiving rabies post-exposure prophylaxis, and inform local animal control or public health officials of bite incidents (26–28). However, the programs in Sri Lanka and Pakistan were limited geographically and relied on bite victims to seek medical care (or a euthanized animal's lab report in the case of Sri Lanka) to trigger data input and case investigation (26, 28). This approach could be prone to under-detection of rabies cases (human and animal), as it is suspected that many dog bite victims do not seek medical care nor animal diagnosis after a potential rabies exposure (8). The REACT app is designed for programs that focus on community-based surveillance and risk-counseling with bite victims. This approach has been shown to increase rabies case detection and improve PEP adherence, both of which contribute to a reduction in human rabies cases and improved program cost-effectiveness (8, 17).

Implementation of REACT was not without difficulties, which primarily were attributed to infrastructural challenges that are common in low- and middle-income countries. Lack of access to electricity and internet were cited by most of the investigators. The REACT app was designed with these challenges in mind and is able to collect and store data locally (on the device) in the absence of internet. At a time when internet is available, data can be automatically or manually uploaded to a cloud-based server. Technology continues to evolve rapidly, including more reliable and low-cost tablets and longer battery life. Issues related to the performance of the app were rare, suggesting that general improvements in information and technology systems will only hasten the speed at which programs adopt app-based electronic health systems.

The evaluation and comparison presented here are subject to at least four limitations. First, this analysis did not evaluate year-to-year variation which would account for variations during the evolution of the program (e.g., improved efficiency, trainings, number of staff) and epidemiology over time. Second, responses to surveys were not anonymous and surveys were administered by the national program manager. While some respondents might not have felt comfortable answering, this is thought to be unlikely since use of the app, although encouraged, was optional. Third, when evaluating the length of time investigators reported to complete the paper investigation form (prior to the app's use) compared to entering data in the app, over half of interviewees reported taking notes on paper during an investigation and later entering the data into the app. While the survey asked how many minutes the paper form took to complete prior to existence of the app, respondents were not asked how many minutes were spent “taking notes” prior to app data entry. Therefore, the time required to complete the app may be under-reflected in this analysis. Correspondingly, respondents were not asked to explain why they took paper notes prior to entering data into the app. Finally, cost per death averted is subject to change based on epidemiologic factors that are difficult to control. As such cost per investigation is the more appropriate measure for comparing programs during different time frames.

## Conclusion

IBCM in Haiti is an effective community-based surveillance system that provides a framework and guidance for bite case investigations and decreases human mortality from rabies. Adoption of the REACT app in Haiti has resulted in improved data quality and completeness, more efficient data reporting and analysis, and higher levels of user acceptability. Rabies endemic countries could refer to Haiti's eIBCM as a cost-effective means to reduce human rabies mortality and improve data consistency and transparency, even in the face of social, political, economic, and natural disruptions.

## Data availability statement

The raw data supporting the conclusions of this article will be made available by the authors, without undue reservation.

## Ethics statement

The studies involving human participants were reviewed and approved by the CDC not to be research, as the purpose of these activities was to investigate and assess a condition of public health importance. CDC Institutional Review Board (IRB) review was not required under the following provision: Public health surveillance activities, 45 CFR 46.102(l)(2). Written informed consent for participation was not required for this study in accordance with the national legislation and the institutional requirements. The animal study was reviewed and approved by CDC Institutional Animal Care and Use Committee (IACUC). Written informed consent for participation was not obtained from the owners because the purpose of this surveillance program is to monitor the occurrence of animal rabies cases in Haiti through the investigation of animal bites and through the testing of pathology samples from deceased animals. The surveillance program is a component of the national rabies control program, which includes vaccination programs, PEP guidelines, and animal quarantine and euthanasia policies. Surveillance will be used to inform ongoing practice within the national rabies control program. As an activity designed to monitor the occurrence of disease in a defined population, as well as to provide feedback to inform ongoing public health practice, this activity is consistent with the attributes of non-research public health practice, as described in current CDC policy.

## Author contributions

RW and AG conceptualized the project. RW acquired funding and supervised the project. RW, CS, YR, and SB synthesized and analyzed study data. AG provided resources and software programming. PD, KC, NF, and HJ managed coordination/execution of activities. CS wrote original draft with review and editing by RW. All authors contributed to the article, reviewed, and approved the submitted version.
