# Corrigendum: Electronic application for rabies management improves surveillance, data quality, and investigator experience in Haiti

**DOI:** 10.3389/fvets.2023.1204839

**Published:** 2023-05-09

**Authors:** Caroline A. Schrodt, Pierre Dilius, Andrew D. Gibson, Kelly Crowdis, Natael Fénelon, Yasmeen Ross, Sarah Bonaparte, Luke Gamble, Frederic Lohr, Haïm C. Joseph, Ryan M. Wallace

**Affiliations:** ^1^National Center for Emerging and Zoonotic Infectious Diseases, Centers for Disease Control and Prevention, Atlanta, GA, United States; ^2^Epidemic Intelligence Service, CDC, Atlanta, GA, United States; ^3^Haiti Ministry of Agriculture, Rural Development and Natural Resources, Port au Prince, Haiti; ^4^Mission Rabies, Cranborne, Dorset, United Kingdom; ^5^The Royal Dick School of Veterinary Studies and The Roslin Institute, University of Edinburgh, Edinburgh, United Kingdom; ^6^Christian Veterinary Mission, Port au Prince, Haiti; ^7^Pan American Health Organization, Port au Prince, Haiti

**Keywords:** rabies, Haiti, integrated bite case management, one health, surveillance, electronic application, tablet, smartphone

In the published article, there was an error regarding the affiliation for Haïm C. Joseph. Instead of having affiliation [2], they should have had [3].

In the published article, there was an error in the author list, and authors Luke Gamble, Frederic Lohr were erroneously excluded. The corrected author list and appears below.

Caroline A. Schrodt^1, 2*^, Pierre Dilius^3^, Andrew D. Gibson^4, 5^, Kelly Crowdis^6^, Natael Fénelon^7^, Yasmeen Ross^1^, Sarah Bonaparte^1^, Luke Gamble^4^, Frederic Lohr^4^, Haïm C. Joseph^3^ and Ryan M. Wallace^1^

In the published article, there was an error. Luke Gamble and Frederic Lohr should have been included in the Conflict of interest and Author contributions sections.

A correction has been made to the Conflict of Interest section. This sentence previously stated:

“AG was project lead for the development of the electronic application as a part of employment for Mission Rabies.

The remaining authors declare that the research was conducted in the absence of any commercial or financial relationships that could be construed as a potential conflict of interest.”

The corrected sentence appears below:

“AG was project lead for the development of the electronic application, with LG and FL supporting the development, as a part of their employment for Mission Rabies.

The remaining authors declare that the research was conducted in the absence of any commercial or financial relationships that could be construed as a potential conflict of interest.”

A correction has been made to the Author Contributions section. This sentence previously stated:

“RW and AG conceptualized the project. RW acquired funding and supervised the project. RW, CS, YR, and SB synthesized and analyzed study data. AG provided resources and software programming. PD, KC, NF, and HJ managed coordination/execution of activities. CS wrote original draft with review and editing by RW. All authors contributed to the article, reviewed, and approved the submitted version.”

The corrected sentence appears below:

“RW, AG, LG, and FL conceptualized the project. RW acquired funding and supervised the project. RW, CS, YR, and SB synthesized and analyzed study data. AG, LG and FL provided resources and software programming. PD, KC, NF, and HJ managed coordination/execution of activities. CS wrote original draft with review and editing by RW. All authors contributed to the article, reviewed, and approved the submitted version.”

The authors apologize for this error and state that this does not change the scientific conclusions of the article in any way. The original article has been updated.

