# Supplementary material for: Electronic application for rabies management improves surveillance, data quality, and investigator experience in Haiti
Source: Front Vet Sci. 2023 Mar 31;10:1052349. doi: 10.3389/fvets.2023.1052349 (PMC10103903; doi:10.3389/fvets.2023.1052349)
Supplement: Supplementary file 1 [file Data_Sheet_1.PDF]

SUPPLEMENTARY MATERIALS

- Table of Contents

  - Page 2: Table A. Risk Assignments of Discordant Animal Case Status Assignments
  - Pages 2-7: IBCM Staff Survey 1.0 (For staff who have used both paper and app)
  - Pages 7-12: IBCM Staff Survey 2.0 (For staff who have ONLY used the APP.)
  - Pages 13-22: Sample Monthly Report
  - Analyses (Separate Excel Documents)
    - Haiti eIBCM Economic Analysis
    - Haiti pIBCM Economic Analysis

Table A. Risk Assignments of Discordant Animal Case Status Assignments

Investigator Assignment	% (No.)
Correct	94.5 (11526/12194)
Incorrect	5.5 (668/12194)
Over-stated risk*	70.8 (473/668)
Understated risk†	29.2 (195/668)

*Over-stated interim risk

†Understated interim risk

IBCM Staff Survey 1.0 (For staff who have used both paper and app)

(Note to interviewer: instructions for the interviewer are italicized in parenthesis and do not need to be read aloud. Please fill out questions 1-4 prior to starting the survey.)

Demographics/Background

1. What is the respondent's first name? ____________________________

2. What is the respondent's last name? ____________________________

3. Is the respondent male or female?
   a. Male
   b. Female

4. What is the respondent's role in the program?
   a. Investigator
   b. Department manager
   c. National program manager
   d. CDC
   e. Other, specify: ____________________________

(Note to interviewer: Please read the following introductory statement aloud. Then you may continue the survey by reading the questions and the answer choices aloud.)

You have been asked to participate in this survey as part of a rabies IBCM surveillance evaluation in Haiti from 2013-2019. We are describing implementation of the electronic IBCM app and comparing the electronic IBCM app to traditional paper forms. The intent of this survey is to learn about your training, use, and opinion of the electronic integrated bite case management (IBCM) "app" that is used to guide bite investigations and collect data for rabies surveillance.

5. What is your age in years? _____

6. What is your highest level of education completed? (select all that apply)
   a. Veterinary Technician

b. Veterinary Agent
c. Other: ____________________

7. Do any teenagers live at your home?
    a. Yes
    b. No

8. In what month and year did you start performing rabies investigations on behalf of the MARNDR Rabies Surveillance Program?
    MONTH ___________ YEAR: ___________

Technology Background + App Training/Use + Paper use

9. On average, how many minutes per week do you spend using a **personal** smart phone, **personal** tablet, or **personal** computer during your free time, tan lib, only? This should NOT include time using these electronics for work purposes. ______

10. In general, how confident do you feel using a smart phone, tablet, or computer?
    a. Very confident
    b. Confident
    c. Neither confident nor unconfident
    d. Not very confident
    e. Not at all confident

11. Acceptability: Have you used the electronic IBCM app in the last month?
    a. Yes
    b. No, I used paper. Explain: _____________
    c. No, I have not investigated cases. Explain: _____________

12. In what month and year did you complete electronic IBCM app training?

    MONTH ______ YEAR: _________

13. After your training, approximately how many investigations did you conduct with the electronic IBCM app before you felt confident in using it correctly? ________________ (Write "not confident" if respondent is still not confident.) ***

14. Did anyone else **not** part of MARNDR, such as friends, clients, neighbors, or family members, ever help you use the electronic IBCM app?
    a. Yes
    b. No

15. On average, how many minutes per week do you use the electronic IBCM app? ______

16. On average, how many minutes does it take to record a complete investigation in the electronic IBCM app? __________

17. Do you enter data directly into the tablet at the same time you are investigating the case(s), or do you hand write data first and then later enter data into the app after the case investigation is completed? ***

18. On average, how many minutes does it take to record a complete investigation using the paper form? __________ ***

We want to learn how your experience with the electronic IBCM app compares to the prior IBCM system that used paper forms. For the following questions please indicate whether you strongly agree, agree, are neutral, disagree, or strongly disagree with each statement.

19. The electronic IBCM app makes it **easier to submit** my case investigations to my supervisor, compared to the paper forms.
    a. Strongly Agree
    b. Agree
    c. Neither agree nor disagree
    d. Disagree
    e. Strongly disagree

20. The electronic IBCM app allows me to submit my case investigations **faster to my supervisor**, compared to the paper forms.
    a. Strongly Agree
    b. Agree
    c. Neither agree nor disagree
    d. Disagree
    e. Strongly disagree

21. The electronic IBCM app allows me to collect **more information** during my case investigations, compared to the paper forms.
    a. Strongly Agree
    b. Agree
    c. Neither agree nor disagree
    d. Disagree
    e. Strongly disagree

22. The electronic IBCM app helps me to be certain that I conduct a **more complete** case investigation, compared to the paper forms.
    a. Strongly Agree
    b. Agree
    c. Neither agree nor disagree

   d. Disagree
   e. Strongly disagree

23. The information collected on the electronic IBCM app helps me understand and assess the **rabies risk** during an investigation, compared to the paper forms. ***
    a. Strongly agree
    b. Agree
    c. Neither agree nor disagree
    d. Disagree
    e. Strongly disagree

24. The electronic IBCM app determines and displays **the case status** of the animal (confirmed, probable, suspect, non-case). Compared to the paper forms, this helps me during the case investigation to understand the rabies risk and recommend PEP to bite victims.
    a. Strongly Agree→ Please specify________________
    b. Agree→ Please specify________________
    c. Neither agree nor disagree
    d. Disagree
    e. Strongly disagree

25. The electronic IBCM app helps me appropriately **determine quarantine periods** for animals that are under investigation, compared to the paper forms.
    a. Strongly Agree→ Please specify________________
    b. Agree→ Please specify________________
    c. Neither agree nor disagree
    d. Disagree
    e. Strongly disagree

26. The app helps me to ensure that investigation results are **communicated** to **all** bite victims, compared to the paper forms.
    a. Strongly Agree → Please specify________________
    b. Agree→ Please specify________________
    c. Neither agree nor disagree
    d. Disagree
    e. Strongly disagree

27. The electronic IBCM app allows for **faster data analysis** compared to paper forms.
    a. Strongly Agree
    b. Agree
    c. Neither agree nor disagree
    d. Disagree
    e. Strongly disagree

****

28. The training I received **prepared me to use** the electronic IBCM app.
    a. Strongly Agree
    b. Agree
    c. Neither agree nor disagree
    d. Disagree
    e. Strongly disagree
    f. I did not receive training for the electronic IBCM app

29. I feel **confident** using the electronic IBCM app *at present*.
    a. Strongly Agree
    b. Agree
    c. Neither agree nor disagree
    d. Disagree
    e. Strongly disagree

(Please note the answer choices have changed for the following questions. Please read all the answer choices aloud to the respondent.)

30. How frequently do you have problems using the electronic IBCM app?
    a. Always
    b. Often
    c. Sometimes
    d. Rarely
    e. Never (If question 24 is Never, skip to question 26.)

We want to learn about problems you've experienced with the electronic IBCM app. The answer choices are as follows:
i. never, ii. rare; iii. sometimes; iv: often

31. In the last 3 months, what problems accessing the electronic IBCM app have you experienced? For each problem, I would also like to know how frequently you experienced these problems.

(Please select all that apply and for each problem encountered, write the roman numeral indicating frequency on the line that follows.)

a. ☐ No internet or data connection
    a. i. never, ii. rare; iii. sometimes; iv: often
b. ☐ Tablet lost/stolen/damaged/unavailable--
    a. i. never, ii. rare; iii. sometimes; iv: often
c. ☐ Tablet nonfunctioning
    a. i. never, ii. rare; iii. sometimes; iv: often
d. ☐ Electronic IBCM app nonfunctioning
    a. i. never, ii. rare; iii. sometimes; iv: often
e. ☐ Dead battery
    a. i. never, ii. rare; iii. sometimes; iv: often
f. ☐ No charger

a. i. never, ii. rare; iii. sometimes; iv: often

g. ☐ I do not know how to use the application

a. i. never, ii. rare; iii. sometimes; iv: often

h. ☐ I do not know how to use the tablet

a. i. never, ii. rare; iii. sometimes; iv: often

i. ☐ Other: please specify ____________________ *(Remember to ask about frequency for each problem listed.)*

a. i. never, ii. rare; iii. sometimes; iv: often

****

32. Can you name three things that you find most frustrating about the app, compared to the paper form?
    a. ______________________________
    b. ______________________________
    c. ______________________________

33. Can you name three things that you find most helpful about the app, compared to the paper form?
    a. ______________________________
    b. ______________________________
    c. ______________________________

34. I would recommend that MARNDR continue to use the app for the national rabies surveillance program
    a. Agree
    b. Disagree

35. With which of the following statements do you most agree?
    a. I prefer to use the paper because it is easier to fill out the form
    b. I prefer to use the paper because the data is more complete
    c. I prefer to use the app because it collects more information
    d. I prefer to use the app because it has features that make investigating rabid animals easier
    e. I have no preference between the app or paper form

IBCM Staff Survey 2.0 (For staff who have ONLY used the APP.)

(Note to interviewer: instructions for the interviewer are italicized in parenthesis and do not need to be read aloud. Please fill out questions 1-4 prior to starting the survey.)

Demographics/Background

1. What is the respondent's first name? ____________________________

2. What is the respondent's last name? ____________________________

3. Is the respondent male or female?
    a. Male
    b. Female

4. What is the respondent's role in the program?
    a. Investigator
    b. Department manager
    c. National program manager
    d. CDC
    e. Other, specify: ___________________________

(Note to interviewer: Please read the following introductory statement aloud. Then you may continue the survey by reading the questions and the answer choices aloud.)

You have been asked to participate in this survey as part of a rabies IBCM surveillance evaluation in Haiti from 2013-2019. We are describing implementation of the electronic IBCM app and comparing the electronic IBCM app to traditional paper forms used previously. The intent of this survey is to learn about your training, use, and opinion of the electronic integrated bite case management (IBCM) "app" that is used to guide bite investigations and collect data for rabies surveillance.

5. What is your age in years? _____

6. What is your highest level of education completed? (select all that apply)
    a. Veterinary Technician
    b. Veterinary Agent
    c. Other: _____________________

7. Do any teenagers live at your home?
    a. Yes
    b. No

8. In what month and year did you start performing rabies investigations on behalf of the MARNDR Rabies Surveillance Program?
    MONTH ___________ YEAR: ___________

Technology Background + App Training/Use

9. On average, how many minutes per week do you spend using a **personal** smart phone, **personal** tablet, or **personal** computer during your free time, tan lib, only? This should NOT include time using these electronics for work purposes. ______

10. In general, how confident do you feel using a smart phone, tablet, or computer?
    a. Very confident
    b. Confident
    c. Neither confident nor unconfident

    d. Not very confident
    e. Not at all confident

11. Acceptability: Have you used the electronic IBCM app in the last month?
    a. Yes
    b. No, I have not investigated cases. Explain: _____________

12. In what month and year did you complete electronic IBCM app training?

    MONTH ______ YEAR: _________

13. After your training, approximately how many investigations did you conduct with the electronic IBCM app before you felt confident in using it correctly? ________________ (Write "not confident" if respondent is still not confident.) ***

14. Did anyone else **not** part of MARNDR, such as friends, clients, neighbors, or family members, ever help you use the electronic IBCM app?
    a. Yes
    b. No

15. On average, how many minutes per week do you use the electronic IBCM app? ______

16. On average, how many minutes does it take to record a complete investigation in the electronic IBCM app? __________

17. Do you enter data directly into the tablet at the same time you are investigating the case(s), or do you hand write data first and then later enter data into the app after the case investigation is completed? ***

We want to learn more about your experience with the electronic IBCM app. For the following questions please indicate whether you strongly agree, agree, are neutral, disagree, or strongly disagree with each statement.

18. The electronic IBCM app makes it **easy to submit** my case investigations to my supervisor.
    f. Strongly Agree
    g. Agree
    h. Neither agree nor disagree
    i. Disagree
    j. Strongly disagree

19. The electronic IBCM app allows me to submit my case investigations **quickly to my supervisor**.
    f. Strongly Agree
    g. Agree
    h. Neither agree nor disagree

i. Disagree
j. Strongly disagree

20. The electronic IBCM app allows me to collect **a lot of information** during my case investigations, compared to the paper forms.
    f. Strongly Agree
    g. Agree
    h. Neither agree nor disagree
    i. Disagree
    j. Strongly disagree

21. The electronic IBCM app helps me to be certain that I conduct a **complete** case investigation.
    f. Strongly Agree
    g. Agree
    h. Neither agree nor disagree
    i. Disagree
    j. Strongly disagree

22. The information collected on the electronic IBCM app helps me understand and assess the **rabies risk** during an investigation. ***
    a. Strongly agree
    b. Agree
    c. Neither agree nor disagree
    d. Disagree
    e. Strongly disagree

23. The electronic IBCM app determines and displays **the case status** of the animal (confirmed, probable, suspect, non-case). This helps me during the case investigation to understand the rabies risk and recommend PEP to bite victims.
    f. Strongly Agree→ Please specify________________
    g. Agree→ Please specify________________
    h. Neither agree nor disagree
    i. Disagree
    j. Strongly disagree

24. The electronic IBCM app helps me appropriately **determine quarantine periods** for animals that are under investigation.
    f. Strongly Agree→ Please specify________________
    g. Agree→ Please specify________________
    h. Neither agree nor disagree
    i. Disagree
    j. Strongly disagree

25. The app helps me to ensure that investigation results are **communicated** to **all** bite victims.

f. Strongly Agree → Please specify_______________
g. Agree→ Please specify_______________
h. Neither agree nor disagree
i. Disagree
j. Strongly disagree

26. The electronic IBCM app allows for **fast data analysis**.
    f. Strongly Agree
    g. Agree
    h. Neither agree nor disagree
    i. Disagree
    j. Strongly disagree

****

27. The training I received **prepared me to use** the electronic IBCM app.
    g. Strongly Agree
    h. Agree
    i. Neither agree nor disagree
    j. Disagree
    k. Strongly disagree
    l. I did not receive training for the electronic IBCM app

28. I feel **confident** using the electronic IBCM app *at present*.
    f. Strongly Agree
    g. Agree
    h. Neither agree nor disagree
    i. Disagree
    j. Strongly disagree

(Please note the answer choices have changed for the following questions. Please read all the answer choices aloud to the respondent.)

29. How frequently do you have problems using the electronic IBCM app?
    f. Always
    g. Often
    h. Sometimes
    i. Rarely
    j. Never (If question 24 is Never, skip to question 26.)

We want to learn about problems you've experienced with the electronic IBCM app. The answer choices are as follows:
i. never, ii. rare; iii. sometimes; iv: often

30. In the last 3 months, what problems accessing the electronic IBCM app have you experienced? For each problem, I would also like to know how frequently you experienced these problems.

(Please select all that apply and for each problem encountered, write the roman numeral indicating frequency on the line that follows.)

j. ☐ No internet or data connection
   a. i. never, ii. rare; iii. sometimes; iv: often
k. ☐ Tablet lost/stolen/damaged/unavailable--
   a. i. never, ii. rare; iii. sometimes; iv: often
l. ☐ Tablet nonfunctioning
   a. i. never, ii. rare; iii. sometimes; iv: often
m. ☐ Electronic IBCM app nonfunctioning
   a. i. never, ii. rare; iii. sometimes; iv: often
n. ☐ Dead battery
   a. i. never, ii. rare; iii. sometimes; iv: often
o. ☐ No charger
   a. i. never, ii. rare; iii. sometimes; iv: often
p. ☐ I do not know how to use the application
   a. i. never, ii. rare; iii. sometimes; iv: often
q. ☐ I do not know how to use the tablet
   a. i. never, ii. rare; iii. sometimes; iv: often
r. ☐ Other: please specify ____________________ *(Remember to ask about frequency for each problem listed.)*
   a. i. never, ii. rare; iii. sometimes; iv: often

****

31. Can you name three things that you find most frustrating about the app?
    a. _____________________________
    b. _____________________________
    c. _____________________________

32. Can you name three things that you find most helpful about the app?
    a. _____________________________
    b. _____________________________
    c. _____________________________

33. I would recommend that MARNDR continue to use the app for the national rabies surveillance program
    a. Agree
    b. Disagree

34. With which of the following statements do you most agree?
    a. I prefer to use the app because it collects a lot of information
    b. I prefer to use the app because it has features that make investigating rabid animals easy

Sample Monthly Report:

Example Rabies Control - Month Report

Report month: April 20XX

Graph of total case investigations this month by Department and Case Status outcome:

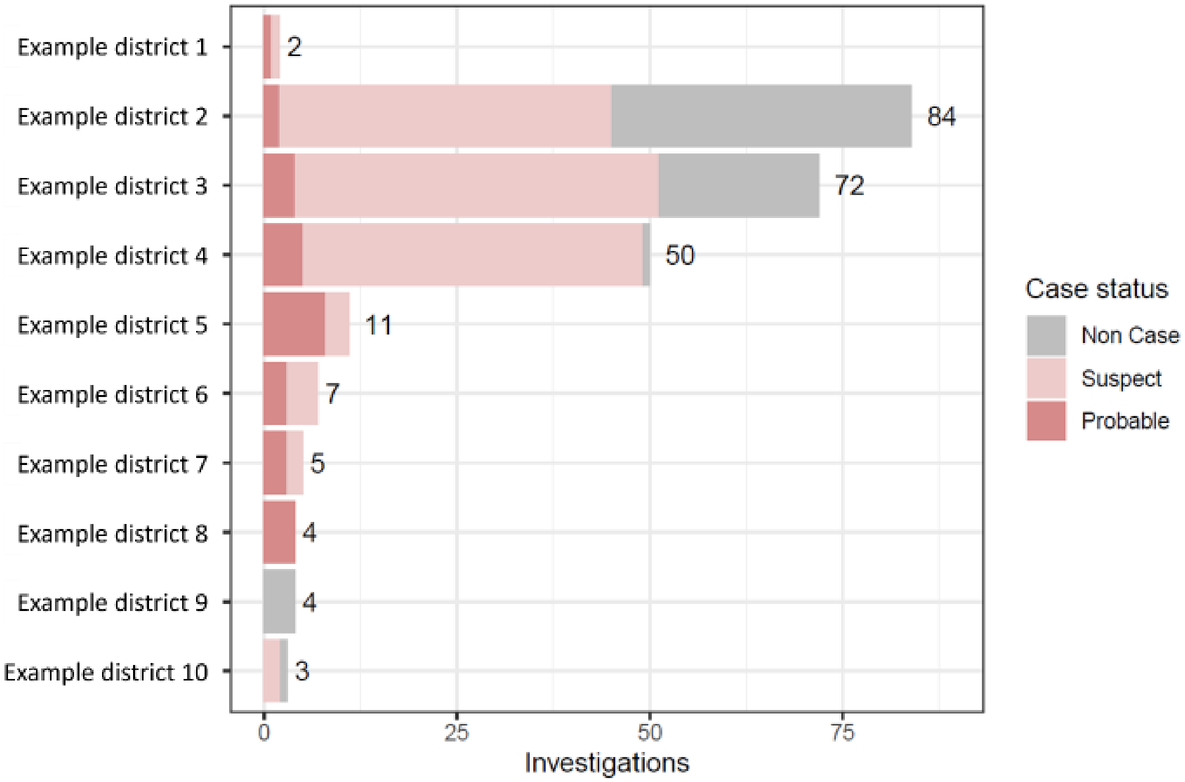

Department	Non Case	Suspect	Probable	Department total
Example district 3	21	47	4	**72**
Example district 10	1	2	0	**3**
Example district 9	4	0	0	**4**
Example district 6	0	4	3	**7**
Example district 8	0	0	4	**4**
Example district 7	0	2	3	**5**
Example district 2	39	43	2	**84**
Example district 4	1	44	5	**50**
Example district 5	0	3	8	**11**
Example district 10	0	1	1	**2**
**NATIONAL TOTAL**	**66**	**146**	**30**	**242**

Maps of investigations and cases this month (April 20XX)

Investigations by Commune

Labels give Department total investigations

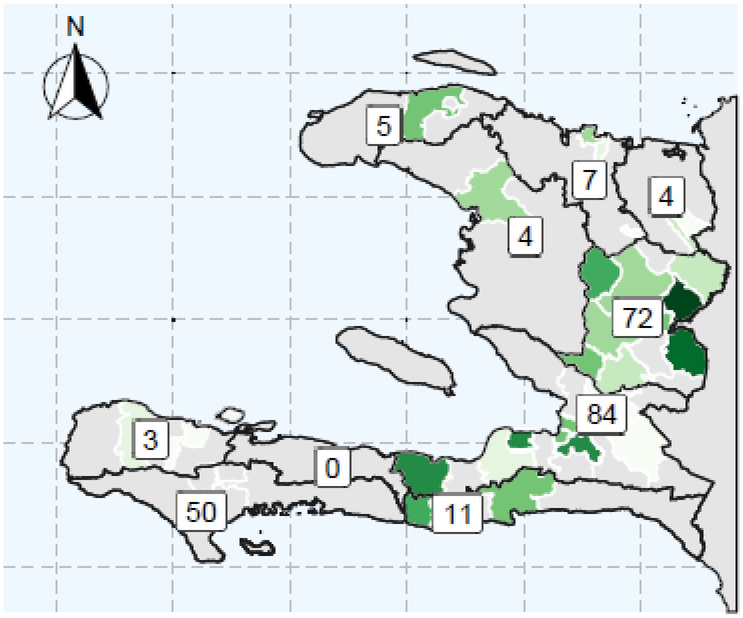

Probable & confirmed cases by Commune

Points are case locations

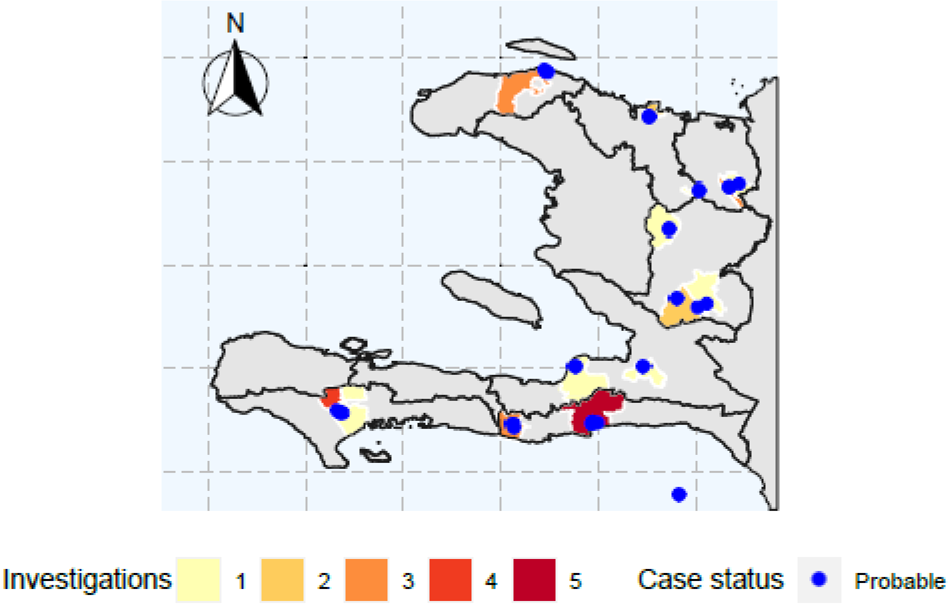

Details of Confirmed and Probable cases for the month of April 20XX

Date	User	App ID	Case ID	Lab ID	Case status	Department	Arrondissement	Commune	Lon	Lat
yyyy-mm-dd	User XX	12345	UID example	NA	Probable	Example	Example	Example	Example	Example
yyyy-mm-dd	User XX	12345	UID example	NA	Probable	Example	Example	Example	Example	Example
yyyy-mm-dd	User XX	12345	UID example	NA	Probable	Example	Example	Example	Example	Example
yyyy-mm-dd	User XX	12345	UID example	NA	Probable	Example	Example	Example	Example	Example
yyyy-mm-dd	User XX	12345	UID example	NA	Probable	Example	Example	Example	Example	Example
yyyy-mm-dd	User XX	12345	UID example	NA	Probable	Example	Example	Example	Example	Example
yyyy-mm-dd	User XX	12345	UID example	NA	Probable	Example	Example	Example	Example	Example
yyyy-mm-dd	User XX	12345	UID example	NA	Probable	Example	Example	Example	Example	Example
yyyy-mm-dd	User XX	12345	UID example	NA	Probable	Example	Example	Example	Example	Example
yyyy-mm-dd	User XX	12345	UID example	NA	Probable	Example	Example	Example	Example	Example
yyyy-mm-dd	User XX	12345	UID example	NA	Probable	Example	Example	Example	Example	Example
yyyy-mm-dd	User XX	12345	UID example	NA	Probable	Example	Example	Example	Example	Example
yyyy-mm-dd	User XX	12345	UID example	NA	Probable	Example	Example	Example	Example	Example
yyyy-mm-dd	User XX	12345	UID example	NA	Probable	Example	Example	Example	Example	Example
yyyy-mm-dd	User XX	12345	UID example	NA	Probable	Example	Example	Example	Example	Example
yyyy-mm-dd	User XX	12345	UID example	NA	Probable	Example	Example	Example	Example	Example
yyyy-mm-dd	User XX	12345	UID example	NA	Probable	Example	Example	Example	Example	Example
yyyy-mm-dd	User XX	12345	UID example	NA	Probable	Example	Example	Example	Example	Example
yyyy-mm-dd	User XX	12345	UID example	NA	Probable	Example	Example	Example	Example	Example
yyyy-mm-dd	User XX	12345	UID example	NA	Probable	Example	Example	Example	Example	Example
yyyy-mm-dd	User XX	12345	UID example	NA	Probable	Example	Example	Example	Example	Example
yyyy-mm-dd	User XX	12345	UID example	NA	Probable	Example	Example	Example	Example	Example
yyyy-mm-dd	User XX	12345	UID example	NA	Probable	Example	Example	Example	Example	Example
yyyy-mm-dd	User XX	12345	UID example	NA	Probable	Example	Example	Example	Example	Example
yyyy-mm-dd	User XX	12345	UID example	NA	Probable	Example	Example	Example	Example	Example
yyyy-mm-dd	User XX	12345	UID example	NA	Probable	Example	Example	Example	Example	Example
yyyy-mm-dd	User XX	12345	UID example	NA	Probable	Example	Example	Example	Example	Example
yyyy-mm-dd	User XX	12345	UID example	NA	Probable	Example	Example	Example	Example	Example
yyyy-mm-dd	User XX	12345	UID example	NA	Probable	Example	Example	Example	Example	Example
yyyy-mm-dd	User XX	12345	UID example	NA	Probable	Example	Example	Example	Example	Example

Rabies ALERT map Communes labelled as on Alert have had a probable or confirmed rabies case within the past 3 months.

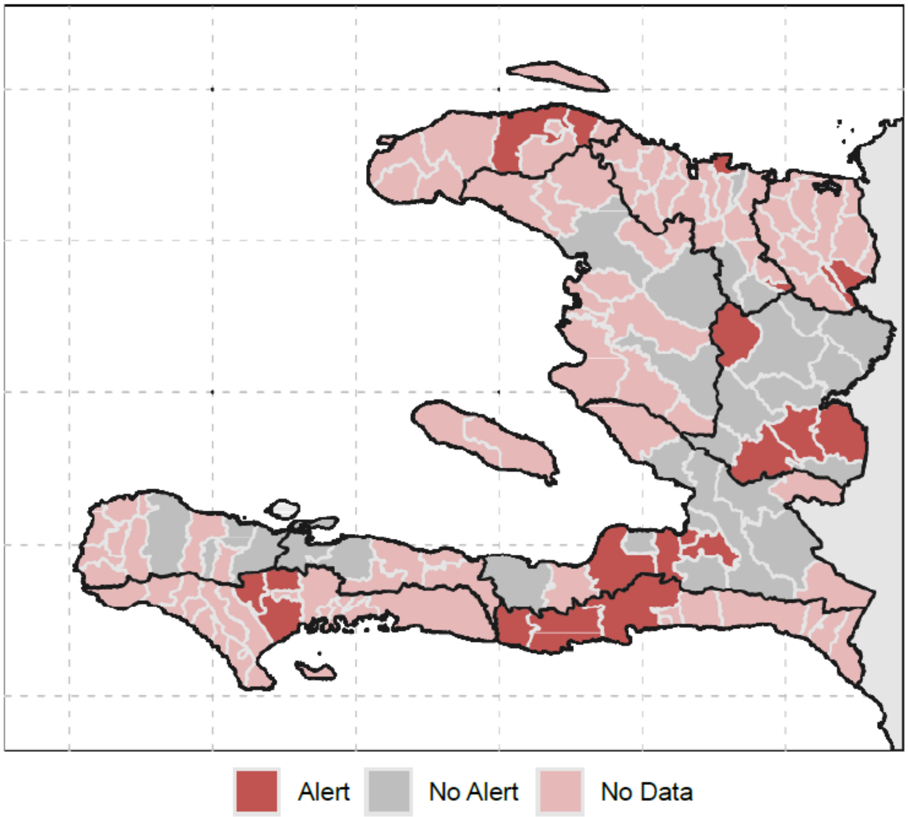

National case investigations and case status outcome per month since recording began (top) and per week for 20[redacted] (bottom)

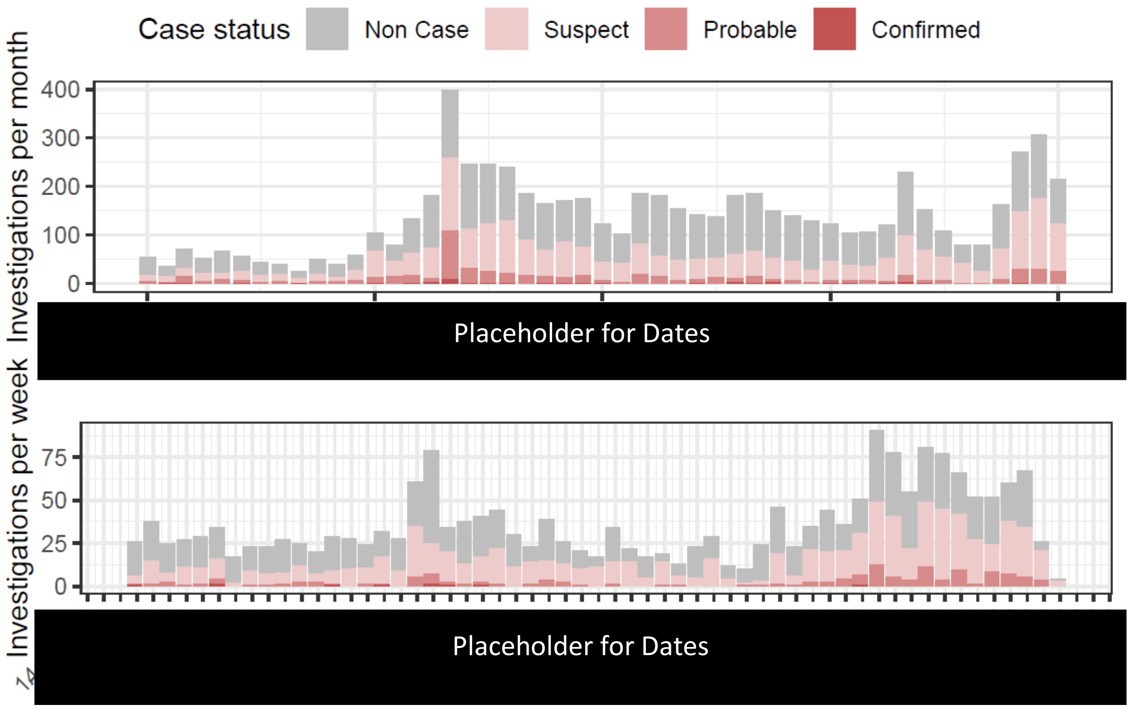

Case outcomes by department
(Numeric text show the number of 'Confirmed' cases per month in each Department)

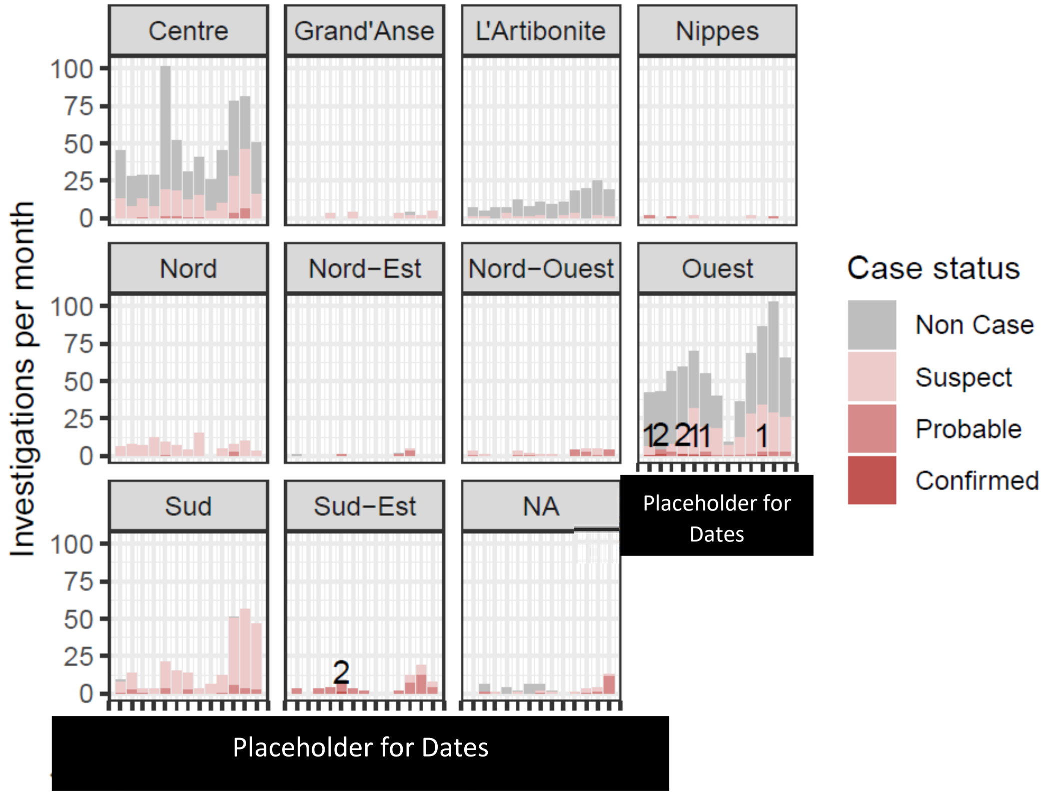

Investigation outcomes for investigations in the month of Feb 20[redacted]

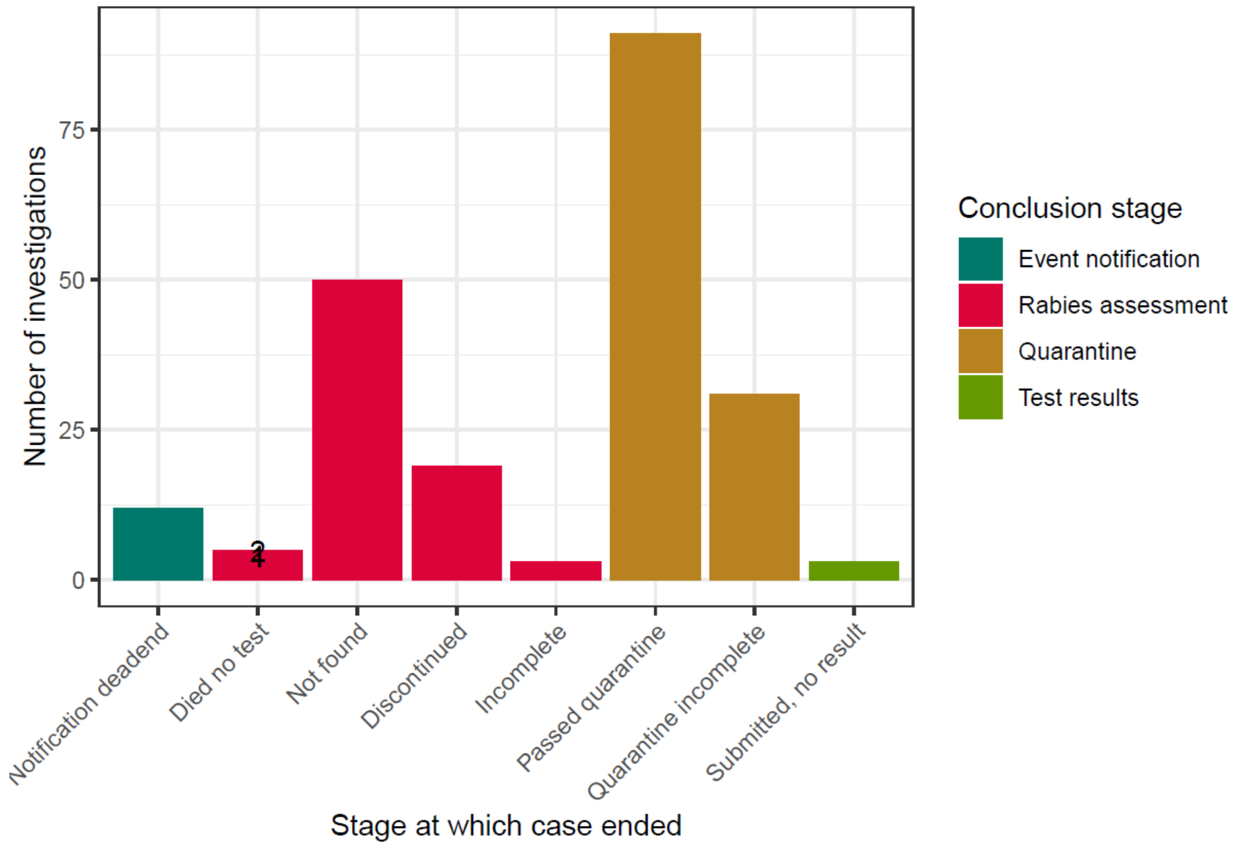

Table of monthly laboratory submissions and case outcomes

Month beginning	Positive	Negative	No result	NA	Total
20[redacted]-01-01	1	0	0	0	**1**
20[redacted]-02-01	2	2	0	0	**4**
20[redacted]-03-01	0	2	0	0	**2**
20[redacted]-04-01	2	1	0	0	**3**
20[redacted]-05-01	3	2	0	0	**5**
20[redacted]-06-01	1	0	0	1	**2**
20[redacted]-07-01	0	0	1	1	**2**
20[redacted]-08-01	0	0	0	1	**1**
20[redacted]-11-01	1	0	3	0	**4**
20[redacted]-12-01	0	0	2	0	**2**
**Year total**	**10**	**7**	**6**	**3**	**26**

Details of all cases reported as submitted for laboratory testing this year.
Cases highlighted in dark red were tested **positive**.
Cases in highlighted in light red were were reported as submitted for testing, but had no result reported in REACT.

Investigation ID	Lab ID	Investigation date	Lab report date	IBCM officer	Department	Arrondissement	Commune	Latitude	Longitude	Final diagnosis	Tests performed	DFA result	DRIT result	PCR result	LFA result	Pending result status	Case status
[redacted]	[redacted]	[redacted]	[redacted]	[redacted]	Ouest	Port-au-Prince	Delmas	[redacted]	[redacted]	positive	dfa	positive	NA	NA	NA	results_complete	test_pos
[redacted]	[redacted]	[redacted]	[redacted]	[redacted]	Ouest	Port-au-Prince	Carrefour	[redacted]	[redacted]	negative	drit	NA	negative	NA	NA	results_pending	test_neg
[redacted]	[redacted]	[redacted]	[redacted]	[redacted]	Ouest	Port-au-Prince	Carrefour	[redacted]	[redacted]	negative	drit	NA	negative	NA	NA	results_pending	test_neg
[redacted]	[redacted]	[redacted]	[redacted]	[redacted]	Ouest	Port-au-Prince	Port-au-Prince	[redacted]	[redacted]	positive	drit	NA	positive	NA	NA	results_pending	test_pos
[redacted]	[redacted]	[redacted]	[redacted]	[redacted]	Ouest	Port-au-Prince	Port-au-Prince	[redacted]	[redacted]	positive	drit	NA	positive	NA	NA	results_pending	test_pos
[redacted]	[redacted]	[redacted]	[redacted]	[redacted]	Ouest	Port-au-Prince	Pétion-Ville	[redacted]	[redacted]	negative	dfa	negative	NA	NA	NA	results_complete	test_neg
[redacted]	[redacted]	[redacted]	[redacted]	[redacted]	Ouest	Léogâne	Léogâne	[redacted]	[redacted]	positive	NA	NA	NA	NA	NA	NA	test_pos
[redacted]	[redacted]	[redacted]	[redacted]	[redacted]	Ouest	Port-au-Prince	Port-au-Prince	[redacted]	[redacted]	positive	drit	NA	positive	NA	NA	results_pending	test_pos
[redacted]	[redacted]	[redacted]	[redacted]	[redacted]	L'Artibonite	les Gonaïves	Gonaïves	[redacted]	[redacted]	negative	drit	NA	negative	NA	NA	results_pending	test_neg
[redacted]	[redacted]	[redacted]	[redacted]	[redacted]	Centre	Hinche	Hinche	[redacted]	[redacted]	negative	NA	NA	NA	NA	NA	NA	test_neg
[redacted]	[redacted]	[redacted]	[redacted]	[redacted]	Sud-Est	Bainet	Bainet	[redacted]	[redacted]	positive	dfa	positive	NA	NA	NA	results_pending	test_pos
[redacted]	[redacted]	[redacted]	[redacted]	[redacted]	Sud-Est	Bainet	Bainet	[redacted]	[redacted]	negative	dfa	negative	NA	NA	NA	results_pending	test_neg
[redacted]	[redacted]	[redacted]	[redacted]	[redacted]	Sud-Est	Jacmel	Jacmel	[redacted]	[redacted]	positive	dfa	positive	NA	NA	NA	results_complete	test_pos
[redacted]	[redacted]	[redacted]	[redacted]	[redacted]	Ouest	Port-au-Prince	Port-au-Prince	[redacted]	[redacted]	negative	drit	NA	negative	NA	NA	results_pending	test_neg
[redacted]	[redacted]	[redacted]	[redacted]	[redacted]	Ouest	Port-au-Prince	Grenier	[redacted]	[redacted]	positive	drit	NA	positive	NA	NA	results_pending	test_pos
[redacted]	[redacted]	[redacted]	[redacted]	[redacted]	Ouest	Léogâne	Léogâne	[redacted]	[redacted]	positive	drit	NA	positive	NA	NA	results_complete	test_pos
[redacted]	[redacted]	[redacted]	[redacted]	[redacted]	Ouest	Port-au-Prince	Delmas	[redacted]	[redacted]	not_tested	drit	NA	not_tested	NA	NA	results_complete	NA
[redacted]	[redacted]	[redacted]	[redacted]	[redacted]	Ouest	Port-au-Prince	Port-au-Prince	[redacted]	[redacted]	not_tested	drit	NA	not_tested	NA	NA	results_complete	NA
[redacted]	[redacted]	[redacted]	[redacted]	[redacted]	Ouest	Port-au-Prince	Pétion-Ville	[redacted]	[redacted]	not_tested	drit	NA	not_tested	NA	NA	results_complete	NA
[redacted]	[redacted]	[redacted]	[redacted]	[redacted]	Centre	Mirebalais	Mirebalais	[redacted]	[redacted]	NA	NA	NA	NA	NA	NA	NA	event_notification_end
[redacted]	[redacted]	[redacted]	[redacted]	[redacted]	Ouest	Port-au-Prince	Pétion-Ville	[redacted]	[redacted]	NA	NA	NA	NA	NA	NA	NA	submitted_no_result
[redacted]	[redacted]	[redacted]	[redacted]	[redacted]	Nord-Est	Vallières	Carice	[redacted]	[redacted]	NA	NA	NA	NA	NA	NA	NA	submitted_no_result
[redacted]	[redacted]	[redacted]	[redacted]	[redacted]	Sud	les Cayes	Camp Perrin	[redacted]	[redacted]	NA	NA	NA	NA	NA	NA	NA	submitted_no_result
[redacted]	[redacted]	[redacted]	[redacted]	[redacted]	Sud-Est	Jacmel	Jacmel	[redacted]	[redacted]	NA	NA	NA	NA	NA	NA	NA	submitted_no_result
[redacted]	[redacted]	[redacted]	[redacted]	[redacted]	NA	NA	NA	[redacted]	[redacted]	NA	NA	NA	NA	NA	NA	NA	submitted_no_result
